# Neutron Larmor diffraction on powder samples

**DOI:** 10.1107/S160057671901611X

**Published:** 2020-02-01

**Authors:** Thomas Keller, Piotr Fabrykiewicz, Radosław Przeniosło, Izabela Sosnowska, Bernhard Keimer

**Affiliations:** a Max Planck Institute for Solid State Research, Stuttgart, Germany; b Max Planck Society Outstation at the Forschungsneutronenquelle Heinz Maier-Leibnitz (FRM-II), Garching, Germany; c Faculty of Physics, University of Warsaw, Poland

**Keywords:** neutron Larmor diffraction, neutron spin–echo, polycrystalline samples, powder diffraction

## Abstract

A hitherto unrecognized resolution effect in neutron Larmor diffraction (LD) is reported, resulting from small-angle neutron scattering (SANS) in the sample. Small distortions of the neutron trajectories by SANS give rise to a blurring of the Bragg angles of the order of a few hundredths of a degree, leading to a degradation of the momentum resolution. This effect is negligible for single crystals but may be significant for polycrystalline or powder samples. A procedure is presented to correct the LD data for the parasitic SANS. The latter is accurately determined by the SESANS technique (spin–echo small-angle neutron scattering), which is readily available on Larmor diffractometers. The analysis technique is demonstrated on LD and SESANS data from α-Fe_2_O_3_ powder samples. The resulting *d*-spacing range agrees with experimental data from high-resolution synchrotron radiation powder diffraction on the same sample.

## Abstract 2

A method to analyse neutron Larmor diffraction data from polycrystalline samples is presented.

## 1. Introduction   

Neutron Larmor diffraction (LD) is a high-resolution technique which permits the measurement of lattice spacings *d_hkl_* and their range Δ*d_hkl_*. The latter arises, for example, from microstrains, magnetostriction, or structural and magnetic domains, or from a small splitting of Bragg peaks resulting from distortions of the crystal lattice. The resolution of current Larmor diffractometers is ∼10^−6^ (relative) of the lattice spacing. Further improvement by one order of magnitude is possible by an optimized instrument design.

The present work is related to a review of the symmetry properties and subsequent revision of the crystal structures of numerous compounds. Synchrotron radiation (SR) powder diffraction studies of the multiferroic BiFeO_3_ (Sosnowska *et al.*, 2012 ▸), CaCO_3_, calcite (Przeniosło *et al.*, 2016 ▸), the transition metal oxides V_2_O_3_, Al_2_O_3_ and Cr_2_O_3_ (Fabrykiewicz *et al.*, 2018 ▸), and hematite (α-Fe_2_O_3_) (Przeniosło *et al.*, 2014 ▸) have shown mono­cli­nic symmetry instead of the previously established trigonal one. Similar studies have shown distortions of the cubic symmetry of chromium (Przeniosło *et al.*, 2018 ▸) and MnO (Fabrykiewicz & Przeniosło, 2016 ▸). The monoclinic distortion observed in α-Fe_2_O_3_ is of the order of Δ*d*/*d* ≃ 2 × 10^−4^, *i.e.* close to the detection limit of SR powder diffraction methods. The LD experiments in this work were conducted in order to confirm this small lattice distortion with a complementary technique and thus to validate the SR data.

LD is based upon the Larmor precession of the neutron spins in parallelogram-shaped magnetic fields along the incident and scattered beams. The geometry of these fields, sometimes labelled precession devices (PDs), was first proposed by Rekveldt (Rekveldt, 2000 ▸; Rekveldt *et al.*, 2001 ▸). Tuning of the field boundaries of the PDs parallel to the diffracting lattice planes yields a Larmor precession phase ϕ_LD_ ∝ *d_hkl_*, where ϕ_LD_ is decoupled from the spectral width and collimation of the neutron beam. The characteristic limitation of conventional diffractometers imposed by the inverse proportionality between intensity and resolution is not effective in LD, and so excellent momentum resolution without excessive loss of intensity is achieved. In this respect, LD is similar to neutron spin–echo, where the energy resolution is decoupled from the monochromaticity of the incident neutron beam.

Experimental studies taking advantage of LD include thermal expansion under hydrostatic and uniaxial pressure (Pfleiderer *et al.*, 2007 ▸; Niklowitz *et al.*, 2010 ▸; Bourdarot *et al.*, 2011 ▸), the formation of structural and antiferromagnetic domains (Náfrádi *et al.*, 2016 ▸), and the splitting of Bragg peaks by lattice distortions (Inosov *et al.*, 2013 ▸; Hu *et al.*, 2015 ▸; Wang *et al.*, 2018 ▸). For thermal expansion only relative changes in the lattice constants are relevant, but LD can also provide absolute values of *d*_*hkl*_ when the instrument is calibrated with a reference monocrystal, such as silicon or germanium. In this way, *d*_*hkl*_ values for polycrystalline Inconel alloys were accurately measured to determine residual stresses (Repper *et al.*, 2010 ▸), and in spin–ice materials the exact values of the lattice constants allowed quantification of the emergent magnetic monopole charge (Ruminy *et al.*, 2016 ▸).

The Larmor diffractometers TRISP at the Heinz Maier-Leibnitz Zentrum (Keller *et al.*, 2002 ▸; Keller & Keimer, 2015 ▸), FLEXX at the Helmholtz-Zentrum Berlin (Groitl *et al.*, 2015 ▸) and ZETA at the Institut Laue–Langevin (Martin *et al.*, 2012 ▸) were originally designed as three-axis spin–echo spectrometers optimized for the spectroscopy of phonons and spin excitations. These instruments take advantage of the resonant neutron spin–echo technique (NRSE) to define the parallelogram-shaped field regions by radio frequency (RF) spin-flip coils (Golub & Gähler, 1987 ▸; Gähler & Golub, 1988 ▸). A recent development uses Wollaston prisms, triangular-shaped DC magnetic fields bounded by superconducting sheets (Li *et al.*, 2017 ▸), as PDs. The advantages of the latter technique are the compact geometry and the increased flexibility achieved by tuning the inclination angles of the magnetic fields by electrical currents, instead of mechanical rotation as in the case of NRSE.

The resolution of LD depends only weakly on the sample mosaic, and for polycrystalline samples no significant reduction in the resolution compared with single crystals is expected. This was confirmed experimentally on polycrystalline aluminium (Rekveldt *et al.*, 2001 ▸) and Inconel alloy (Repper *et al.*, 2010 ▸). If the size of the crystallites is in the sub-micrometre range, small-angle neutron scattering (SANS) of the incident and diffracted beams might lead to a blurring of the Bragg angles. This effect is typically small, with SANS angles of the order of a few hundredths of a degree, and there will be no SANS intensity at the large Bragg angles θ_B_ ≃ 50° used in LD experiments. Initial LD measurements performed with α-Fe_2_O_3_ polycrystalline samples in cylindrical vanadium containers have shown *d*-spacing ranges considerably broader than those obtained with SR diffraction. This broadening results from small-angle scattering in the sample, which is negligible in the SR experiments, as it increases with sample diameter and neutron or X-ray wavelength. Both parameters are more favourable in the case of SR, with a sample diameter (capillary) of 0.5 mm and an X-ray wavelength of 0.4 Å, compared with a sample width of several millimetres and a neutron wavelength of ∼2 Å in LD.

In this paper we discuss the resolution of LD including SANS. The effect can be corrected for, if the SANS scattering probability *S*(θ) is known. Calculation of *S*(θ) is possible, but it requires detailed knowledge of the powder grain morphology and scattering length density profiles. In addition, strong multiple scattering is expected for the large sample diameters of several millimetres typically used in neutron diffraction experiments. Accurate modelling of this multiple scattering is difficult. Thus, a measurement of *S*(θ) is preferable. This can be efficiently performed on the Larmor diffractometer, which is readily converted into a spin–echo small-angle scattering (SESANS) instrument by changing the field polarities and inclination angles of the PDs (Rekveldt, 1996 ▸; Rekveldt *et al.*, 2003 ▸; Keller *et al.*, 1995 ▸). Both LD and SESANS data are then consistently collected on the same sample in one experimental run.

In the following we first review LD including parasitic SANS. We discuss the properties of the SESANS technique and derive a method for correcting LD data for SANS effects. Finally, we work out an example of the analysis technique using experimental data from α-Fe_2_O_3_ powder samples.

## 2. Larmor diffraction including SANS   

In this section we review the principles of LD and then include the effect of SANS.

**Figure 1:**
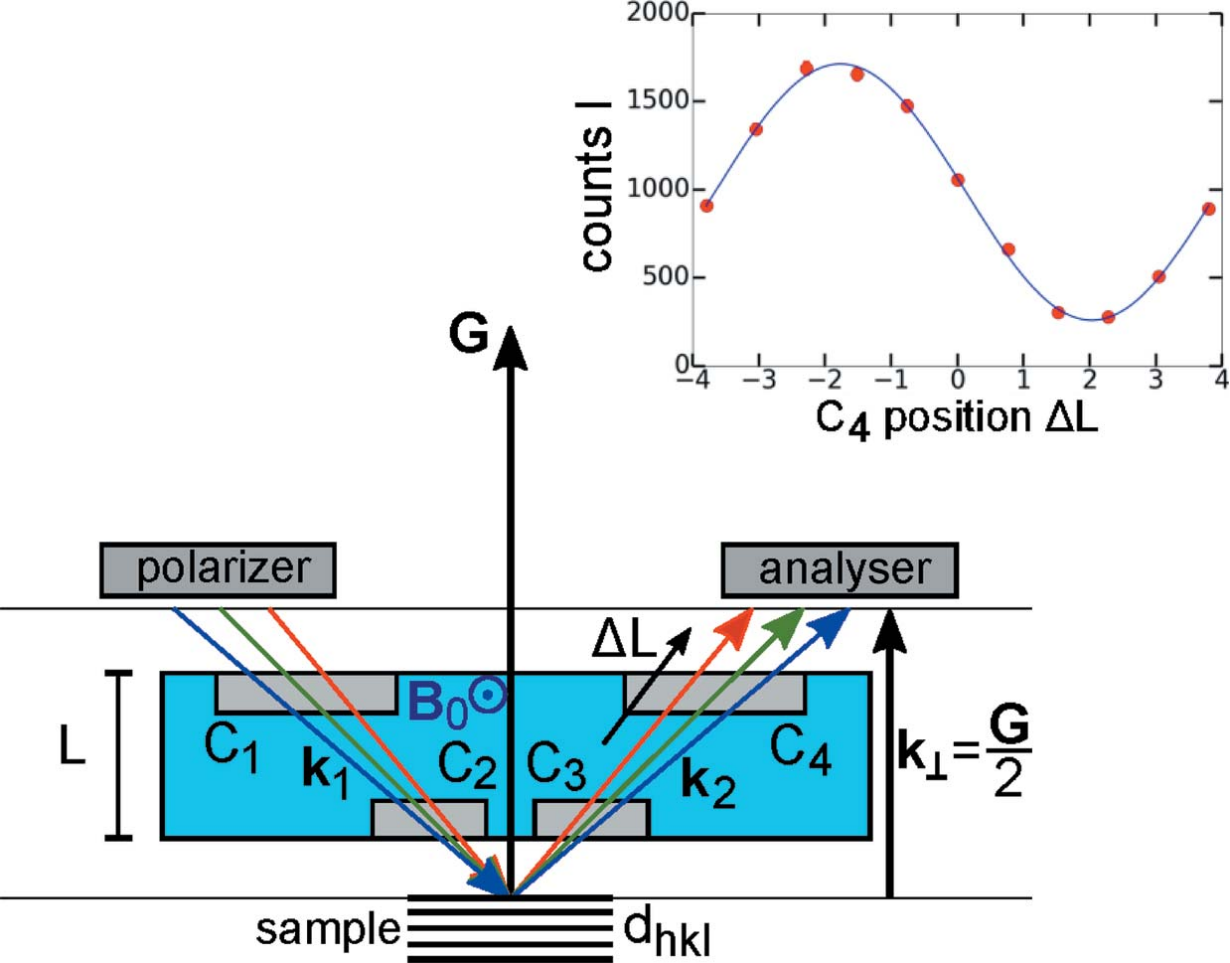
Sketch of a Larmor diffractometer. A polarized neutron beam crosses the uniform field **B**_0_ twice. The boundaries of **B**_0_ are oriented parallel to the lattice planes *d*_*hkl*_, *G* = 2π/*d*_*hkl*_. In the resonant spin–echo configuration (NRSE), instead of a uniform field **B**_0_ four RF spin flippers C_1_–C_4_ define the boundaries of the precession regions, and there is no field along the flight path between the flippers and in the sample region. C1–C4 are double coils, each consisting of two single RF spin-flip coils with opposite field direction (known as the ‘bootstrap’ technique; Gähler & Golub, 1988 ▸). The Larmor phase shift Δϕ_LD_ and the polarization are obtained from the count rate *I* versus the position Δ*L* of the coil C_4_ (Δ*L* in the inset is in units of millimetres): *I*(Δ*L*) = *I*_0_{1 + *P*cos[2π(Δ*L* − Δ*L*_0_)/*L*_per_]}, where the period is *L*_per_ = 2π*v*_n_/ω_L_, *v*_n_ is the neutron velocity and ω_L_ the Larmor frequency. With the offset Δ*L*_0_, the phase is Δϕ_LD_ = 2πΔ*L*_0_/*L*_per_.

Rekveldt proposed the field geometry shown in Fig. 1 ▸, a uniform DC magnetic field **B**_0_ with boundaries oriented parallel to the diffracting lattice planes (Rekveldt, 2000 ▸; Rekveldt *et al.*, 2001 ▸). The neutron spins are initially polarized perpendicular to **B**_0_ and cross the field twice, before and after being diffracted at the lattice planes of a crystal with inter-planar spacing *d*_*hkl*_ and corresponding reciprocal-lattice vector *G* = 2π/*d*_*hkl*_. The Larmor phase ϕ_LD_ = ω_L_ *t*_t_ accumulated by a neutron spin after passing both fields is proportional to the Larmor frequency ω_L_ = γ*B*_0_, with γ = 2π × 2.916 kHz gauss^−1^ and the transition time *t*_t_ = *L*/*v*_⊥_ that the neutron spends in the field. The parameter *v*_⊥_ is the velocity component perpendicular to the field boundary, with *v*_⊥_ = 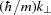, *m* the neutron mass and *k*_⊥_ = *G*/2 = π/*d*_*hkl*_:
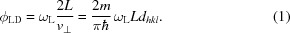
ϕ_LD_ only depends on the field integral *J*_0_ = 2ω_L_ *L* and the lattice spacing *d*_*hkl*_, and is independent of the Bragg angle. The resonant spin–echo technique offers an easy way of defining the required flat-field boundaries by radio-frequency spin–flip coils (Fig. 1 ▸).

A shift in *d*_*hkl*_, for example by thermal expansion, is measured by tracking the phase shift Δϕ(*T*) = ϕ_LD_ ∊_*hkl*_ versus the temperature *T*, where 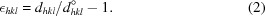


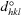
 is the mean or unperturbed value of *d*_*hkl*_. *d*_*hkl*_ is typically spread, for example by the effect of microstrains. Then the phase is also spread as 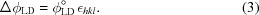


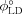
 is related to 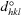 via equation (1). The final beam polarization is given by the average
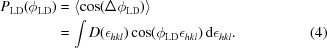

*D*(∊_*hkl*_) is a normalized distribution function. *P*(ϕ_LD_) is measured for a series of values ϕ_LD_(*B*_0_), where for each ϕ_LD_ the position Δ*L* of coil C4 is scanned through one Larmor period as sketched in Fig. 1 ▸. *P* is then obtained from the amplitude of the cosine-shaped *I*(Δ*L*). (The integration limits of all integrals in this section can be chosen as ∓∞.)

For the measurement of thermal expansion it is important to note that Δϕ(*T*) is affected by *D*(∊_*hkl*_), if the latter is asymmetric and if the asymmetry changes with *T*. This might result from an asymmetric splitting of a Bragg peak related to a structural phase transition. In cases where *D*(∊_*hkl*_) is symmetric, even if the width or shape changes with *T*, there will be no effect on Δϕ(*T*).

We now include the distortion of the neutron trajectories by SANS, and the misalignment between the lattice planes and the boundaries of **B**_0_, which necessarily happens for mosaic or polycrystalline samples. Both effects disturb the correlation between the lattice vector *G* and *k*_⊥_, such that *k*_⊥_ 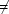 *G*/2. This distortion of the trajectories by SANS leads to an accumulation of additional Larmor phase, resulting in a reduction in *P* in equation (4). The calculation of the phase change depends on whether SANS happens before or after Bragg diffraction. SANS in the incident beam changes the Bragg angle, whereas the path length in the first precession field stays the same, so that the Larmor phase changes. SANS in the diffracted beam does not affect the Bragg angle, but the path length in the second precession field changes.

**Figure 2:**
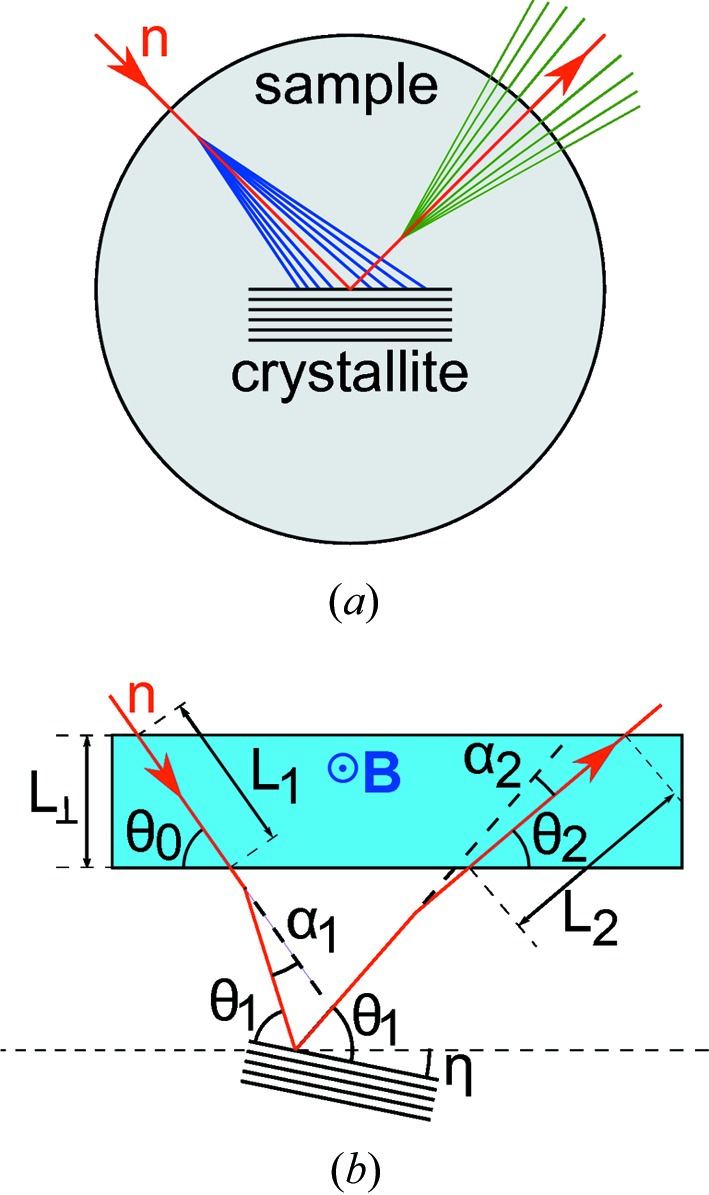
(*a*) SANS in a powder sample before and after Bragg diffraction at a crystallite. (*b*) A single Bragg diffraction event at a crystallite. *L*_1,2_ are the path lengths in the precession field **B**. *L* is the width of the field area **B**_0_. α_1,2_ are the SANS angles. θ_0_ and θ_2_ are the angles of the trajectories with the field boundaries, and θ_1_ is the Bragg angle. η is the angle between the field boundary and the diffracting lattice plane. The signs of θ_0_, θ_1_ and θ_2_ are positive. α_1_, α_2_ and η have a negative sign for clockwise rotation, as shown in the sketch.

We first calculate ϕ_LD_ for single SANS events in the incident and diffracted beams, and then take the average assuming a range of SANS angles, which will be quantitatively determined in a separate SESANS experiment. The geometry is defined in Fig. 2 ▸, with the following relations:
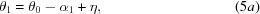


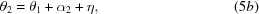


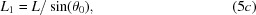


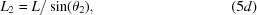


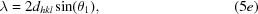


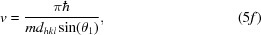


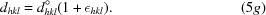
[The last equation is identical to equation (2) and is repeated here for better readability.] *m* and *v* = ℏ*k*/*m* are the neutron mass and velocity, respectively. The Larmor phases ϕ_1,2_ accumulated before and after Bragg diffraction are 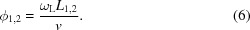
The total phase is 
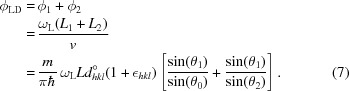
Expanding equation (7) with the definitions of equations (5)[Disp-formula fd5a] to first order in ∊, α_1,2_ and η gives
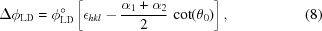
where
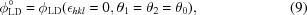


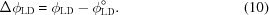
The first-order term in η is zero. The second-order terms of the preceding expansion are summarized in Appendix *A* [App appa]. The leading term is ∼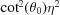, which is negligible for the experimental parameters in this work. The first term in equation (8) is the usual LD phase related to variations in *d*_*hkl*_, while the second term describes the small-angle scattering.

The polarization *P*(ϕ_LD_) is given as a generalization of equation (4) by the average 
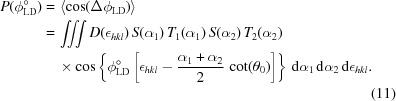

*S*(α_1_) and *S*(α_2_) are the normalized ranges of the SANS angles before and after scattering, respectively, and *T*_1,2_(α_1,2_) are angular transmission functions of the diffractometer, given by the collimations of the incident and scattered beams, respectively. The cosine term in equation (11) splits according to cos(*a* + *b* + c) = cos(*a*) cos(*b*) cos(*c*) + sin…, and the sine terms integrate to zero, as the *S* and *T* distribution functions are symmetric. The polarization is then 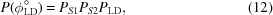


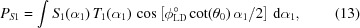


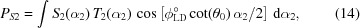
where *P*_LD_ is given by equation (4). The first two factors, 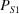 and 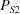, describe the effect of SANS before and after diffraction, respectively. These factors differ in the beam collimation *T*_1,2_ and the width of the small-angle scattering *S*_1,2_. The widths of *S*_1,2_ will typically be much smaller than the widths of *T*_1,2_, such that the latter have only a small influence on the value of the integral. Thus we will simplify *T*_1_ = *T*_2_ = *T*. The widths of *S*_1_ and *S*_2_ are only identical for symmetric scattering configurations, such as cylindrical sample cross sections, or flat samples in a symmetric configuration with respect to *k*_1,2_, and might be quite different for asymmetric sample shapes.

One aim of an LD experiment is to measure the distribution width of the lattice spacing *D*(∊_*hkl*_). To extract *P*_LD_ from the experimental data 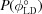 in equation (12), the SANS integrals *P*_*S*1_, *P*_*S*2_ have to be known. In the following section we show that *P_S_* can be determined experimentally by taking advantage of the SESANS technique, and thus for the data analysis there is no need to use an analytical expression for *P_S_*. Nevertheless, an analytical formula is often helpful. As a simple model, we take Gaussians for the distributions *D*, *S*_1,2_ and *T*, with FWHMs of 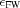, 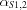 and 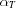, respectively. These Gaussians have the form 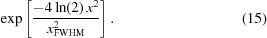
Using the tabulated integral 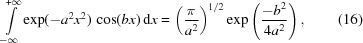
we obtain for the polarization [equation (12)] 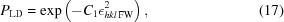


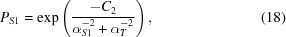


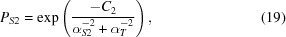


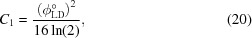


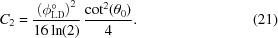



## 3. The SESANS technique   

**Figure 3:**
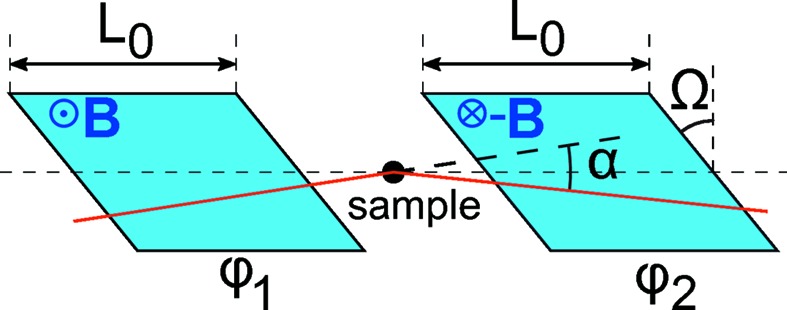
Spin–echo small-angle scattering configuration. The fields *B* of the two precession regions have opposite signs, as opposed to the LD configuration.

We briefly review the principles of SESANS and give scaling formulas for flat and cylindrical samples to convert SESANS data to the LD geometry. SESANS uses two identical field regions (or precession regions defined by RF spin flippers) with opposite signs of the magnetic field, with boundaries inclined by an angle Ω (Fig. 3 ▸). Unscattered neutrons gain equal precession phases in both regions (ϕ_1_ = −ϕ_2_), even if the path is inclined with respect to the central axis. If the neutron is scattered by a small angle α, the path length in the second field changes (Rekveldt, 1996 ▸; Rekveldt *et al.*, 2003 ▸; Keller *et al.*, 1995 ▸): 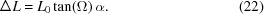
The phase difference Δϕ = ϕ_1_ − ϕ_2_ is 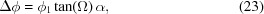
where ϕ_1_ is the Larmor phase accumulated in one precession region, 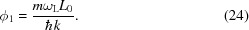
The polarization *P*(ϕ_1_) is given by an integral similar to *P*_*S*1_, *P*_*S*2_ in equation (12): 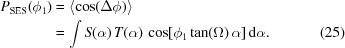
It is not necessary to model *S*(α) for subsequent analysis of the LD data, but it is often convenient to have an analytical expression. As a simple model we again take Gaussian distributions [equation (15)] for *S*(α) and *T*(α) with FWHMs of α_*S*_ and α_*T*_, respectively. Using equation (16) we get 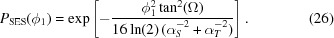
Typically 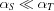, so that the latter is negligible in equation (26).

It is useful to express the SESANS phase [equation (23)] in terms of the spin–echo length *z* and the momentum transfer *Q*: 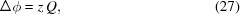
where 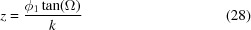
or 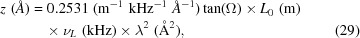
with ν_*L*_ = ω_*L*_/(2π).

**Figure 4:**
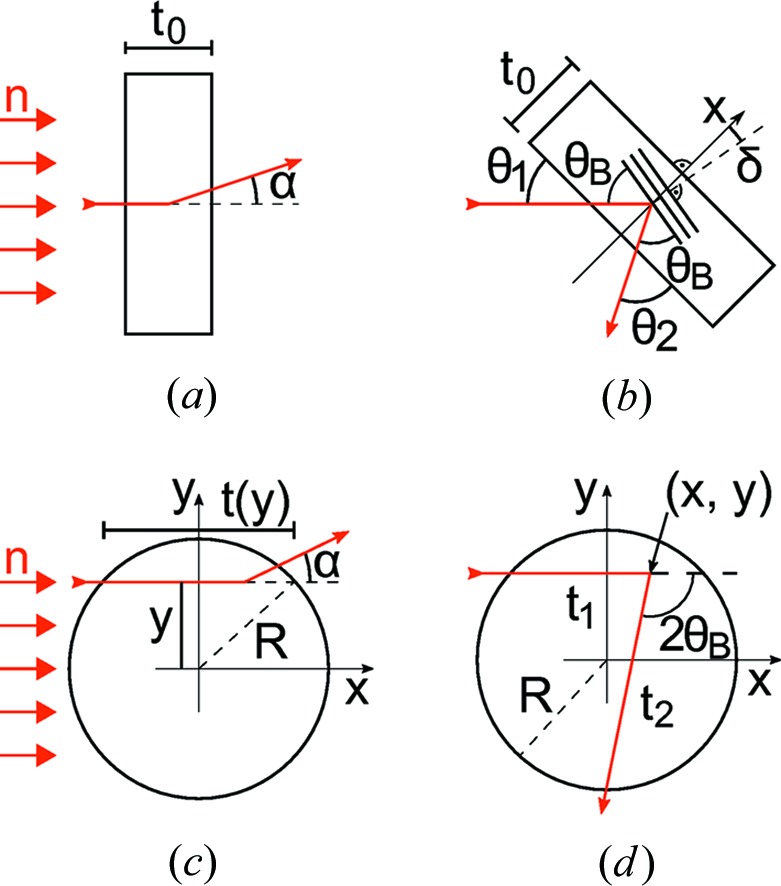
(*a*), (*b*) Rectangular sample. (*a*) In the SESANS configuration, the sample is oriented perpendicular to the incident neutron beam. A scattering event changes the direction of the neutron trajectory by an angle α. (*b*) In the LD configuration, a neutron is Bragg-diffracted at a crystallite located at a distance *x* from the sample face. In the symmetric reflection configuration, the angles of the incident and outgoing neutron trajectories with the sample face are equal to the Bragg angle (θ_1_ = θ_2_ = θ_B_). A small misalignment δ of the sample face leads to θ_1_ = θ_B_ − δ, θ_2_ = θ_B_ + δ. (*c*), (*d*) Cylindrical sample in (*c*) the SESANS and (*d*) the LD configuration.

We now show how 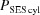 for a cylindrical sample can be calculated using SESANS data 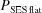 from a flat sample, and vice versa. We will take advantage of a scaling relation for 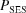 with respect to *k* and the sample width *t* derived by Rekveldt and Andersson (Rekveldt *et al.*, 2003 ▸; Andersson *et al.*, 2008 ▸), which is valid even in the presence of strong multiple scattering:
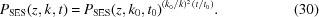
The transformation 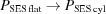 is useful to verify equation (30) experimentally. The transformation 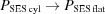 is needed in the next section to analyse LD data collected on a cylindrical sample.


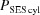
 is obtained from 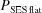 by averaging and scaling the latter for the different path lengths *t*(*y*) of a neutron beam crossing a cylindrical sample [see Figs. 4 ▸(*a*) and 4 ▸(*c*)]. The polarization of individual trajectories is weighted by the transmission Tr = exp[−Σ_*k*_ *t*(*x*)], where Σ_*k*_ and Σ_th_ are the total absorption cross sections for neutrons with wavevectors *k* and *k*_th_ (th for thermal), with *k*_th_ = 2π/λ_th_ and λ_th_ = 1.8 Å. For most isotopes there is 1/*k* scaling if 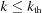: 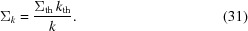
With the path length *t* = 2(*R* ^2^ − *y* ^2^)^1/2^, the width of the flat sample *t*_0_ and the cylinder radius *R* we get
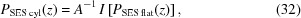


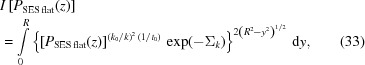


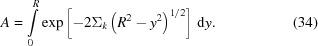
Calculating 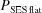 from 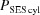 data corresponds to an inversion of equation (32). This is easily achieved numerically by searching for solutions for 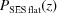 that satisfy the equation 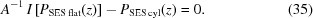

*A* and *I* are the same as in equation (32). Suitable algorithms for finding roots of nonlinear functions, such as fzero() in MATLAB (The MathWorks Inc., Natick, MA, USA), are available in many numerical libraries.

## 4. Scaling of SESANS data to the LD geometry   

In this section, we will determine the integrals *P*_*S*1,2_ in equation (12) from the previously measured SESANS data, for both flat and cylindrical sample geometries. For the flat sample, there are two symmetric configurations, reflection and transmission, with the sample surface perpendicular and parallel to **Q**, respectively. As the effective path length in a flat sample of width *t*_0_ at the Bragg angle θ_B_ is *L*_r_ = *t*_0_/sin(θ) in reflection and *L*_t_ = *t*_0_/cos(θ) in transmission, *L*_t_ > *L*_r_ for θ > 45°. For the LD experiments we used θ = 55°, such that *L*_t_/*L*_r_ ≃ 1.4, leading to increased SANS in the transmission configuration. This configuration is unfavourable in our case and we will not include it in the following discussion.

*P*_*S*1_, *P*_*S*2_ [equation (12)] and *P*_SES_ [equation (25)] are very similar expressions, if the field inclination angles Ω in both SESANS and LD are set to Ω = 90° − θ_B_, so that tan(Ω) = cot(θ_B_). However, the ranges of SANS angles *S*(α) in SESANS and *S*_1,2_(α_1,2_) in LD are not equal, as the geometries for the two experimental configurations are different, and thus the effective neutron path lengths inside the sample are not the same.

In the SESANS configuration [Fig. 4 ▸(*a*)], the faces of the flat sample are aligned perpendicular to the beam. The effective neutron path length corresponds to the sample width *t*_0_. Small variations in the neutron path length resulting from finite beam collimation α_T_ ≃ 1° and from small-angle scattering of the order of *t*_0_/*t* ≃ 1 − cos(α_T_/2) can be neglected in equation (30).

In the LD configuration, the path lengths vary with the location of the diffraction event. For a symmetric setup in reflection geometry as shown in Fig. 4 ▸(*b*), the path lengths of the incident and diffracted neutron trajectories are equal, with *t*_1,2_(*x*) = *x*/sin(θ_1,2_). We obtain the polarization *P*_*S*1_, *P*_*S*2_ by averaging and scaling the SESANS polarization 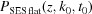 measured for a flat sample of width *t*_0_ according to equation (30). The weight of an individual neutron trajectory is given by the transmission Tr_1,2_(*x*) = exp[−Σ_*k*_ *t*_1,2_(*x*)].
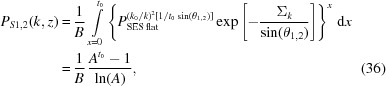


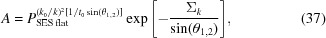


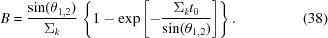



In symmetric reflection geometry with θ_1_ = θ_2_, we get *P*_*S*1_ = *P*_*S*2_. A detuning δ as shown in Fig. 4 ▸(*b*) ▸ increases *t*_1_(*x*) and decreases *t*_2_(*x*), so that the product *P*_*S*1_ × *P*_*S*2_ changes in second order in δ, and a small detuning of the order of a few degrees is not significant.

The cylindrical sample geometry [Figs. 4 ▸(*c*) and 4 ▸(*d*)] is treated in a similar way by taking the average of the SESANS polarization 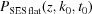 with the transmission as a weight factor. In the case of a cylindrical sample, the SESANS data should be measured on the same sample. Then 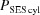 has to be converted to 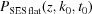 [equation (35)] with a specific but arbitrary *t*_0_. For a neutron diffracted at a crystallite located at (*x*, *y*), the length of the incident trajectory inside a sample of radius *R* is *t*_1_ = *x* + (*R* ^2^ − *y* ^2^)^1/2^.
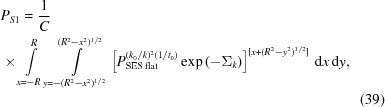
with 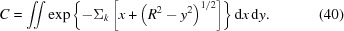
The integration boundaries are the same as in equation (39). For symmetry reasons, *P*_*S*2_ = *P*_*S*1_.

## 5. Experimental   

The LD measurements were conducted on the TRISP spectrometer at the FRM II. TRISP was designed as a thermal three-axis spectrometer incorporating the resonant spin–echo technique for the high-resolution spectroscopy of phonons and spin excitations (Keller *et al.*, 2002 ▸), and it also operates efficiently as a Larmor diffractometer and SESANS instrument. The primary spectrometer includes a V-cavity polarizing neutron guide, a velocity selector and a pyrolytic graphite (002) monochromator. The spin–echo precession devices and the sample are housed in a mu-metal shield with a residual magnetic field of <5 mOe to avoid parasitic spin precession. For the LD measurements, the scattering angle 2θ_B_ was kept constant at 110° and the incident wavevector was varied in the range 2.08 < *k*_i_ < 2.66 Å^−1^ to access various Bragg peaks. In LD mode, the RF coil rotation angles were set to Ω = 90° − θ_B_ = 35° to align the field boundaries parallel to the lattice planes. The same angles Ω = 35° with opposite sign in the second precession field were used in SESANS mode.

The sample was hematite, α-Fe_2_O_3_ (powder), as used in earlier studies (Stekiel *et al.*, 2015 ▸; Fabrykiewicz *et al.*, 2017 ▸). Hematite is a promising material for such studies because of the negligible incoherent neutron scattering of iron and oxygen. The powder was annealed at 1100 K for 5 h in air to reduce internal strain. This annealing step reduced ∊_*hkl*_ by ∼30%. We used two sample cells, a cylindrical vanadium container (labelled ‘cylinder’), inner diameter 12 mm and wall thickness 0.2 mm, and a flat quartz glass cell (labelled ‘flat’), inner dimensions 30 × 30 × 2 mm and 0.5 mm glass width. The flat cell produced an unstructured background (glass peak) in the LD measurement. LD was measured for (024), (116), (214) and (300) at the *k*_i_ values given in Table 1 ▸ using both cylindrical and flat containers.

**Figure 5:**
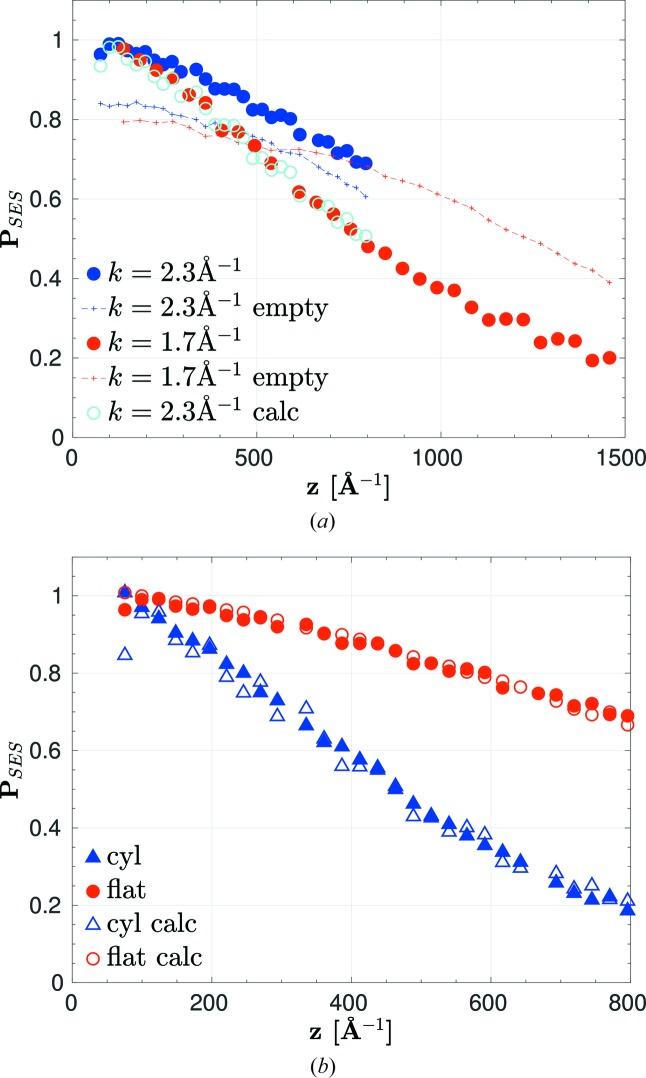
SESANS data for α-Fe_2_O_3_ powder. (*a*) 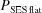 for *k* = 1.7 and 2.3 Å^−1^ for the flat quartz glass container without sample (empty), and with sample normalized by the empty container data. The maximum *z* for a given maximum precession field **B** scales ∼1/*k* ^2^. The blue open circles show the *k* = 2.3 Å^−1^ data scaled to *k* = 1.7 Å^−1^ by equation (30). These scaled data coincide with the experimental data for *k* = 1.7 Å^−1^. (*b*) *P*_SES_ for the flat and cylindrical containers at *k* = 2.3 Å^−1^. 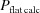 is the polarization calculated for the flat sample geometry by taking the 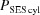 data [equation (35)], and vice versa for 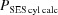 [equation (32)]. The data for the cylindrical (flat) container are plotted as blue triangles (red circles). Solid symbols show measured data, while open symbols are calculations. The error bars in the measured values are smaller than the symbols.

For the SESANS experiments, the scattering angle was set to zero and the neutron beam was shaped by a cadmium mask to the sample size. 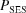 was measured for both empty and filled sample cells at *k* = 2.30 Å^−1^, which is roughly in the centre of the *k* range used for LD, and in addition at *k* = 1.70 Å^−1^ for the flat sample to test the scaling with *k* according to equation (30). All measurements are shown in Fig. 5 ▸, where the data with sample were normalized to the empty cell measurements. (See also Table 1 ▸ for a summary of all data collected on α-Fe_2_O_3_).

**Figure 6:**
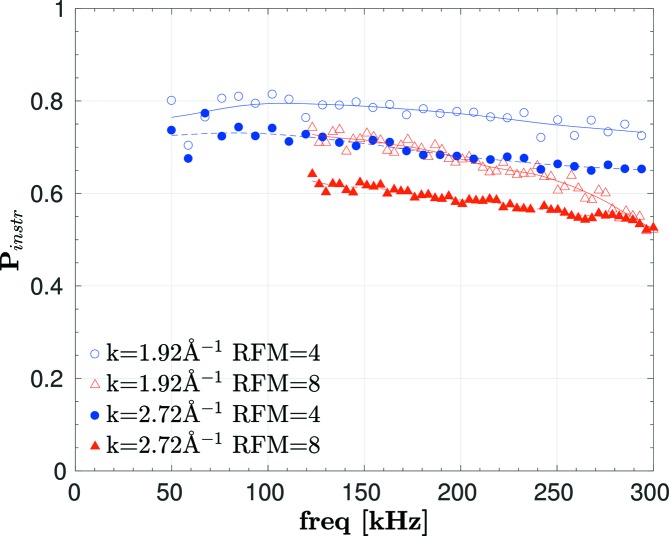
Instrumental resolution in LD mode measured on a Ge monocrystal using the (220) and (004) reflections with *k* = 1.92 Å^−1^ and *k* = 2.72 Å^−1^, respectively. The scattering angle was 110° and the coil inclination angle Ω = 35°. RFM = 4 (8) is the number of active RF coils. The lines are obtained by smoothing the data. Error bars are smaller than the symbols.

In LD mode, first the instrumental resolution *P*_instr_(ϕ_LD_) was determined with a Ge monocrystal as reference sample with a negligible range of *d*_*hkl*_, where for a perfect instrument we expect *P*_instr_ = 1. The instrument configuration for this reference measurement was the same as for the subsequent LD experiment on the powder samples, with fixed 2θ = 110° and Ω = 35°. The Ge (220) and (004) Bragg reflections were accessible at *k* = 1.92 and 2.72 Å^−1^, respectively. Fig. 6 ▸ shows these data for two different operation modes, where one (RFM = 4) or both RF coils (RFM = 8) in the individual coil sets C1–C4 (Fig. 1 ▸) were energized. These two modes differ in the effective Larmor frequency, which is two (four) times the RF for RFM = 4 (8), and they are used to cover the range of low (high) Larmor phase. The *k* dependence of *P*_instr_ is a property of the polarizing neutron guide. The dependence on frequency and RFM, especially the scatter of the data points at low frequency, results from field inhomogeneities in the RF coils and from stray fields in between the coils.

**Figure 7:**
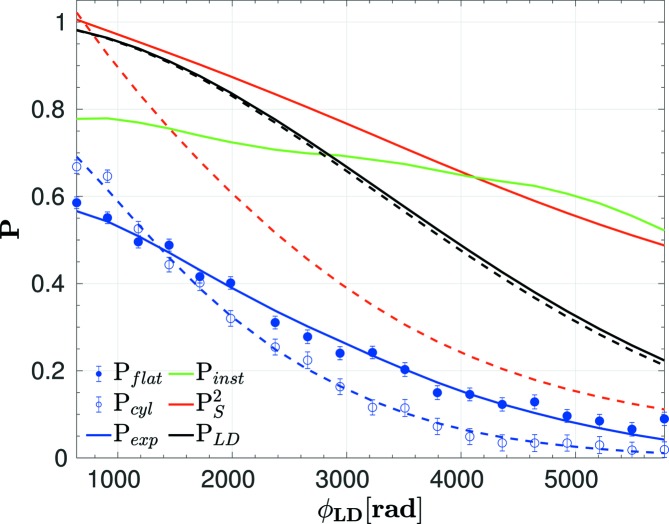
LD data for the (024) reflection of α-Fe_2_O_3_ for the flat quartz glass sample container (width 2 mm, solid symbols, solid lines) and the cylindrical vanadium container (diameter 12 mm, open symbols, dotted lines). The counting time per point was 20 min. *P*_instr_ is the same for both containers. The fit (blue line) to *P*_exp_ is a product of the instrumental *P*_instr_, SANS 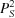 and *P*_LD_ [see equation (41)]. The latter depends on the range of *d*_*hkl*_. A constant factor *P*_0_ takes unpolarized background into account.

Fig. 7 ▸ shows the LD data for the (024) reflection of α-Fe_2_O_3_ measured at *k* = 2.08 Å^−1^ for the flat container (solid lines) and the cylindrical container (dotted lines). Data for the other Bragg peaks are shown in the supporting information. The SANS contribution *P*_*S*_ is much smaller for the flat container. Normalizing the raw data by *P*_*S*_ and by the instrumental polarization *P*_instr_ (identical for both containers) leads to very similar intrinsic *P*_LD_.

The results of LD measurements for α-Fe_2_O_3_ are compared with the results obtained by Stekiel *et al.* (2015 ▸) on the high-resolution SR powder diffractometer ID22 at the ESRF (Fitch, 2004 ▸). The observed SR diffraction peak shape was described using a pseudo-Voigt function. The instrumental contribution of ID22 was estimated by measuring a reference LaB_6_ sample.

## 6. Results and discussion   

In this section we discuss the analysis of LD data from α-Fe_2_O_3_ powder. The analysis steps include (i) determination of the instrumental resolution by means of a monocrystal reference sample, (ii) measurement of the small-angle scattering by the SESANS technique, (iii) scaling these data to the LD geometry including absorption and (iv) determination of *D*(∊_*hkl*_).

In LD, SESANS and other neutron spin–echo techniques, the measured signal, that is the polarization versus the Larmor precession phase, is a product of intrinsic and instrumental effects. This is in contrast with conventional diffraction and spectroscopy, where the measured signals are convolutions of the instrumental resolution and sample-related effects (Mezei, 1980 ▸; Mezei *et al.*, 2003 ▸). This is explained, in brief, by the fact that the polarization is a probability, namely the expectation value of the neutron spinor. The probabilities of instrumental effects, such as non-perfect RF coils, and sample intrinsic effects, such as the range of *D*(∊_*hkl*_), are statistically independent, so that the probabilities (polarizations) are multiplied. A second qualitative argument is that LD and spin–echo techniques provide cosine-Fourier transforms of distribution functions [equations (11) and (25)], such that convolutions are converted to products. In the present case of an LD experiment, the measured polarization *P*_exp_ is
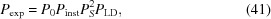
where *P*_*S*_ and *P*_LD_ are defined in equations (12) and (17). A constant factor *P*_0_ accounts for additional effects leading to a loss of polarization, independent of the Larmor phase, including non-polarized background from the sample and spin precession resulting from small randomly distributed ferromagnetic moments in the sample. *P*_inst_ is taken from Fig. 6 ▸ and is independent of the sample. The same argument holds for the SESANS experiment, where 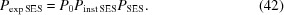
The factor *P*_*S*_ in equation (41) is calculated from 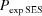 in equation (42) by applying equations (36) and (39) for the flat and cylindrical samples, respectively. The instrumental resolution *P*_inst_ is measured by means of a ‘perfect’ sample with no intrinsic effects, such as a monocrystal with no range of *d*_*hkl*_ in the case of LD, an empty container in the case of SESANS, and a purely elastic scatterer in the case of energy-resolved spin–echo spectroscopy. In the current experiment, we used as reference samples a Ge single crystal for LD or an empty cell for SESANS. *P*_inst_(freq, RFM, *k*) from the Ge crystal is shown in Fig. 6 ▸. The polarization depends on the frequency, the number RFM of energized RF coils, and the wavevector *k*. We use the smoothed curves in Fig. 6 ▸ to average the small parasitic oscillations, and a linear interpolation in *k* between the two data sets taken at *k* = 1.92 and 2.72 Å^−1^. For the following analysis, *P*_inst_(freq, RFM, *k*) has to be converted to *P*_inst_(ϕ_LD_), shown as a line in Fig. 7 ▸. This conversion is ambiguous, as there are two data sets with different RFMs for *P*_inst_. Thus, we always use the data with the same RFM as used for the LD measurement *P*_exp_ of the powder sample.

Fig. 5 ▸ shows SESANS data normalized by the corresponding empty cell scans to eliminate instrumental effects [equation (42)], such as SANS in the coils close to the sample and non-ideal performance of the RF coils. The width α_*S*_ (FWHM) for the small-angle distributions obtained by fitting the Gaussian model equation (26) to the normalized data gives 0.0650 (5), 0.1138 (5) and 0.1427 (5)° for the flat cell at *k* = 2.3 and 1.7 Å^−1^ and for the cylindrical cell at 2.3 Å^−1^, respectively. The parameter α_*S*_ depends strongly on *k* and on the sample width, but is still much smaller than the angular transmission of the instrument α_*T*_ ≃ 1°, so that the latter has no effect in equation (26).

The LD experiment is usually performed at many Bragg reflections, each with a different *k*. The SESANS measurement should at least be done at the lowest of these *k* values. Scaling of the SESANS data to other *k* is possible using equation (30), as is demonstrated in Fig. 5 ▸(*a*) by scaling the *k* = 2.3 Å data to *k* = 1.7 Å. These scaled data coincide with the measured data. Scaling of SESANS data between flat and cylindrical sample shapes by equations (32) and (35) also works, as is shown in Fig. 5 ▸(*b*), where again the scaled and measured data coincide. The absorption of the α-Fe_2_O_3_ powder was included in these plots, although it is small and has no visible effect (σ_th_ = 0.10 cm^−1^ for bulk α-Fe_2_O_3_; the powder density is roughly 40% less). The scaling relation (30) might not hold for unconventional SANS processes, such as multiple Bragg scattering (MBS), which might contribute significantly to the SANS cross section (Barker & Mildner, 2015 ▸). Our analysis technique is also valid for such unconventional SANS processes, if the scaling equation (30) holds. The latter can be experimentally tested by taking SESANS data at several different *k* values.

The LD data for a (024) reflection (*k* = 2.08 Å^−1^) of the α-Fe_2_O_3_ powder are shown in Fig. 7 ▸. This peak was measured at the smallest *k* = 2.08 Å^−1^ and thus has the largest SANS contribution. *P*_*S*_ shows a more pronounced decay for the cylinder due to the increased path length and increased SANS of the cylinder compared with the flat sample. The raw data without any corrections correspond to 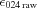 values of 13.5 (5) × 10^−4^ for the cylinder and 8.80 (11) × 10^−4^ for the flat container. Including all corrections, *P*_LD_ is obtained from a fit to equation (17), corresponding to ∊_024_ of 7.1 (2) × 10^−4^ and 7.2 (4) × 10^−4^ for the flat and cylindrical samples, respectively. (The errors are statistical errors from the fit.) This means that, after correction, the values for flat and cylindrical samples agree, where for the flat sample the SANS correction is much smaller. The polarization offsets *P*_0_ are 0.89 (1) for the cylinder and 0.74 (1) for the flat cell, indicating the increased background from the flat quartz glass container. Data for the other Bragg peaks are shown in the supporting information.

The flat sample shape is superior to the cylinder, since for a given beam diameter (typically 20 mm) a larger sample volume at a smaller width can be used. As a rule of thumb, the SANS should be limited to *P*_SES_ 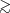 0.5 for the maximum *z* and minimum *k*, as the relative error usually increases with decreasing *P*. This condition is roughly met in the present experiment for the flat sample, but not for the cylinder, although the sample mass is the same in both cases. The only disadvantage of the flat sample is an additional decay of the polarization in the case of an offset δ [Fig. 4 ▸(*b*)] from the symmetric reflection geometry. For the present data, we numerically calculated that for δ < 10° the variation δ∊_024_ < 0.07, *i.e.* only 1/3 of the statistical error. These errors can be avoided by a precise alignment of the sample cell.

**Figure 8:**
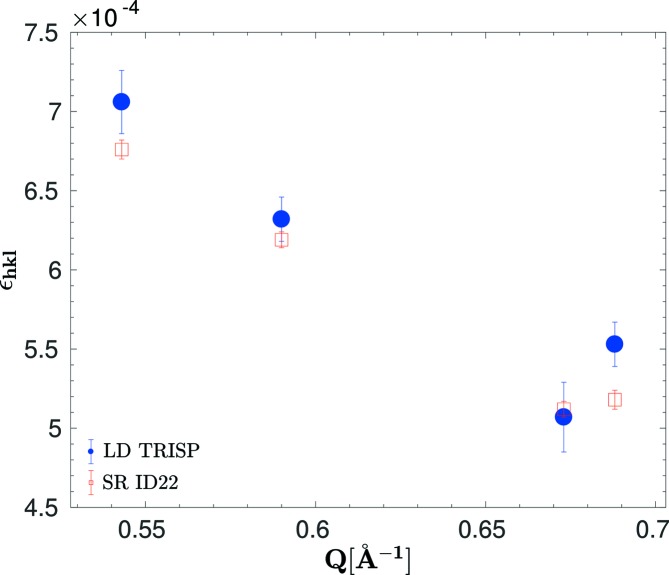
Δ*d*/*d* spacing distribution width ∊_*hkl*_ (FWHM) versus *Q* = 1/*d*_*hkl*_ for four Bragg peaks of α-Fe_2_O_3_ obtained from LD (flat container) and SR diffraction. The data correspond from left to right to the reflections (024), (116), (214) and (300).

In Fig. 8 ▸ the ∊_*hkl*_ obtained by LD for four Bragg peaks (flat sample) are compared with the *d*-spacing distribution widths observed on ID22. The latter were determined by numerical deconvolution of the instrumental resolution function (LaB_6_ reference) from the observed raw-data Bragg peaks. The agreement of SR diffraction with LD confirms our earlier findings about the monoclinic deformation of the α-Fe_2_O_3_ lattice (Przeniosło *et al.*, 2014 ▸). The widths calculated for the monoclinic structure model (Przeniosło *et al.*, 2014 ▸) are ∊_024_ = 2.22 × 10^−4^, ∊_116_ = 1.67 × 10^−4^, ∊_214_ = 0.95 × 10^−4^ and ∊_300_ = 1.12 × 10^−4^. The measured values are much larger, but their variation follows the variation of the calculated values. The increased width is probably due to microstrains in the powder grains.

The measured ∊_*hkl*_ are close to the resolution limit of SR diffraction. For LD, it is not straightforward to define the resolution. Variations in ∊_*hkl*_ are detected as a change in the polarization *P*_LD_. In typical experiments with monocrystalline samples without SANS, the error in ∊_*hkl*_ is close to 10^−5^. The situation is more complicated for powder samples, where in the case of the flat container at the (024) reflection SANS contributes a broadening of 1 × 10^4^ to ∊_*hkl*_. Thus, the statistical errors of the order of ±1.5 × 10^−4^ shown in Fig. 8 ▸ as a result of the fit are also a good estimate for the resolution. The good agreement between LD and SR diffraction validates the related data analysis techniques, *i.e.* the measurement of the instrumental resolution with the LaB_6_ reference sample in SR diffraction and the determination of the instrumental resolution by a monocrystal and the correction for SANS in the case of LD.

## 7. Summary   

In this work we have shown that small-angle scattering makes a significant contribution to the range of *d*_*hkl*_ measured by neutron Larmor diffraction. We have described a quantitative procedure to measure the SANS by means of the spin–echo small-angle neutron scattering technique, and derived scaling expressions to include the SESANS data in the analysis of the LD data collected on flat or cylindrical samples. We have shown that using a flat sample in reflection geometry is preferable to using cylindrical samples, as for a given sample mass the effective neutron path length, and thus the effect of SANS, is minimized for the flat sample. Finally, we have applied the new analysis method to LD data from hematite and calcite powder (for the latter see the supporting information). These LD data confirm the *hkl*-dependent *d*-spacing range previously observed by high-resolution SR diffraction.

## Supplementary Material

Additional SESANS and LD data. DOI: 10.1107/S160057671901611X/in5031sup1.pdf

## Figures and Tables

**Figure 9:**
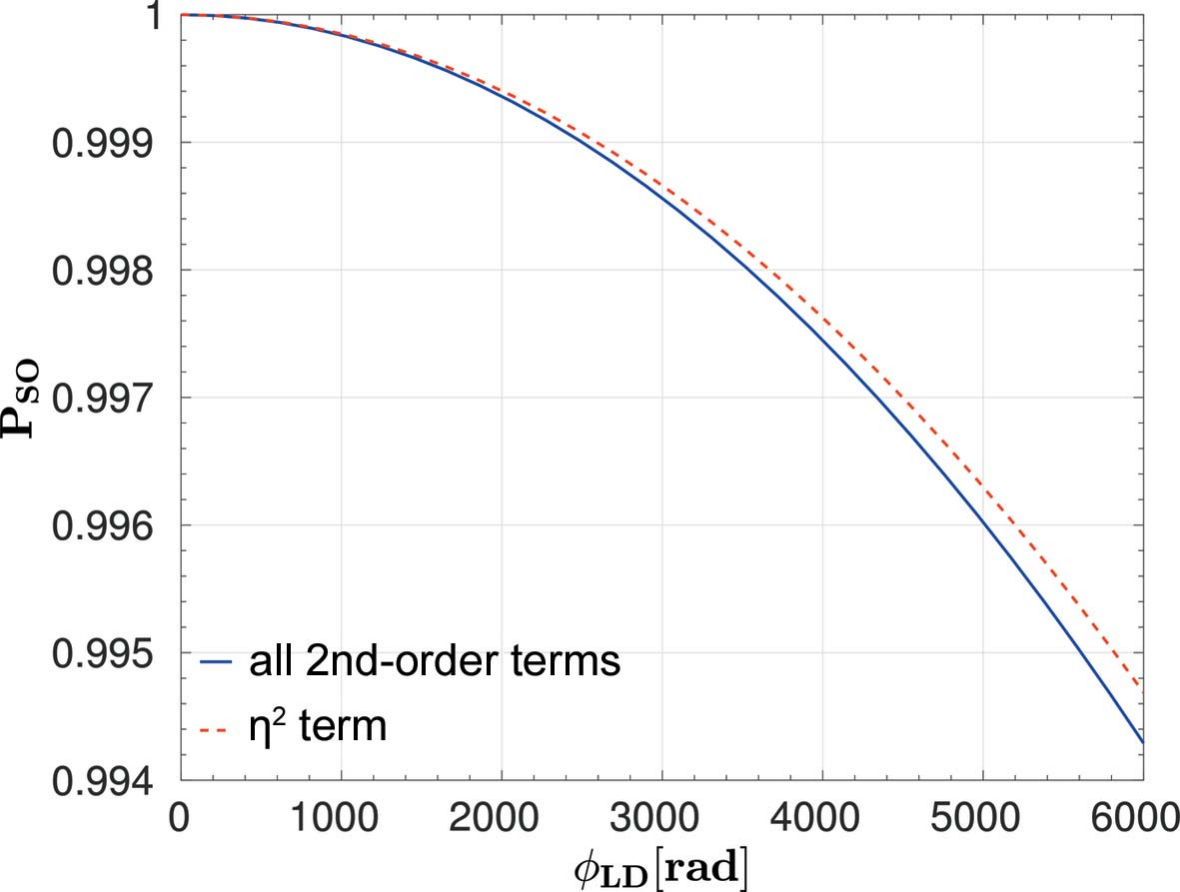
Second-order terms of ϕ_LD_ [equation (46)] and the dominant η^2^ term *P*_33 SO_.

**Table 1:** Experimental data on α-Fe_2_O_3_ powder LD experiments were performed using four Bragg peaks. The flat container was set to reflection geometry.

No.	*k* (Å^−1^)	(*hkl*)	Mode	Container	Contents
1	2.082	(024)	LD	Flat, reflection	Filled
2	2.082	(024)	LD	Cylindrical	Filled
3	2.263	(116)	LD	Flat, reflection	Filled
4	2.263	(116)	LD	Cylindrical	Filled
5	2.581	(214)	LD	Flat, reflection	Filled
6	2.581	(214)	LD	Cylindrical	Filled
7	2.639	(300)	LD	Flat, reflection	Filled
8	2.639	(300)	LD	Cylindrical	Filled
9	1.70		SESANS	Flat	Empty
10	1.70		SESANS	Flat	Filled
11	2.30		SESANS	Flat	Empty
12	2.30		SESANS	Flat	Filled
13	2.30		SESANS	Cylindrical	Empty
14	2.30		SESANS	Cylindrical	Filled

## Appendix A Second-order terms of ϕ_LD_

The preceding discussion was based on the first-order expansion [equation (8)] of ϕ_LD_ in equation (7). Here, we show that the second-order terms of this expansion are negligible for the analysis of the experimental data in the present work.

The expansion of equation (7) to second order in α_1_, α_2_, η and ∊_*hkl*_ gives

with the parameter vector **X** =  and the coefficients

The polarization in equation (41) gains an additional factor *P*_SO_ from the second-order terms:

with

*F*_*i*_(*X*_*i*_) are Gaussian distribution functions for the parameters *X*_*i*_, with FWHM α_*S*1_ = α_*S*2_ = 0.09°, ∊_FW_ = 7 × 10^−4^ and η_FW_ = 0.33°. This last parameter was obtained from the width of the rocking scan of a single perfect crystal with the same spectrometer configuration as used for LD. η_FW_ is smaller than the collimation (∼1°), as the wavelength bands of the incident and diffracted beams are limited by the monochromator and analyser, and thus the range of Bragg angles for a given *d*_*hkl*_ is limited. Calculation of η_FW_ is in principle possible, but a suitable resolution formalism for Larmor diffractometers taking all spectrometer parameters into account, similar to the work of Habicht *et al.* (2003 ▸) and Groitl *et al.* (2018 ▸) for three-axis spin–echo spectrometers, is not yet available.

The second-order terms in equation (43) are very small, except the η^2^ term (∼*M*_33_η^2^). Fig. 9 ▸ shows the polarization equation (46) including all terms, and the η^2^ term *P*_33 SO_ separately, for the experimental parameters of this work. This dominant term changes the resulting range of ∊_*hkl*_ = 7.1 (4) × 10^−4^ by 1.7 × 10^−6^, more than one order of magnitude less than the statistical error in ∊_*hkl*_. Thus we neglected this term in the present analysis. If no monochromator or analyser is used, *P*_33 SO_ is large and should be included in the analysis as an additional factor in equation (41), such that
