# Supplementary material for: Neutron Larmor diffraction on powder samples
Source: J Appl Crystallogr. 2020 Feb 1;53(Pt 1):88–98. doi: 10.1107/S160057671901611X (PMC6998774; doi:10.1107/S160057671901611X)
Supplement: Supplementary file 1 [file j-53-00088-sup1.pdf]

Supplementary Information – Neutron Larmor diffraction on powder samples

T. Keller, P. Fabrykiewicz, R. Przeniosło, I. Sosnowska, and B. Keimer

Here we present additional SESANS and LD data on hematite (α-Fe_2O_3) and calcite ($CaCO_3$) powder samples. Besides the cylindrical vanadium (diameter 12 mm) and the flat quartz glass (width 2 mm) containers we used a flat aluminum container with an inner width of 2 mm and a window thickness if 0.3 mm, both in reflection and transmission geometry.

SESANS and LD experiments on $CaCO_3$ were conducted to get a further test for the LD data analysis procedure including SANS. The calcite powder for the LD experiment was previously well characterized in a high resolution synchrotron radiation (SR) diffraction study [1]. Calcite has a negligible contribution to incoherent neutron scattering.

S1. SESANS

The results of the SESANS experiments are summarized in Table S1. The α_{SESANS} give the Gaussian full-width-at-half-maximum of the angular small angle distribution in degrees. The subscripts *raw* and *norm* indicate raw data and data normalized to the empty container, respectively.

The SANS of the empty containers is in the order of 0.05°, where the cylindrical vanadium container tends to be slightly better than aluminum and quartz glass. The α_{SESANS} increases with decreasing k for both empty and filled containers. For the α-Fe_2O_3 sample, α_{SESANS} of the flat container is reduced by more than a factor 2 compared to the cylindrical container due to the smaller width of the latter.

The smaller α_{SESANS} for $CaCO_3$ results from the increased grain size of $\sim$ 800 nm (α-Fe_2O_3: $\sim$ 400 nm) and from a smaller scattering length density (2/3 of α-Fe_2O_3). The neutron absorption cross section of $CaCO_3$ is by a factor 14.5 smaller than for α-Fe_2O_3 and is thus negligible for our SESANS and LD experiments.

TABLE S1. Summary of all SESANS experiments.

k[Å^{-1}]	sample	container	$\alpha_{\mathrm{SESANS\ raw}}$[°]	$\alpha_{\mathrm{SESANS\ norm}}$[°]
1.7	empty	flat quartz	0.0618(7)	—
1.7	empty	flat Al	0.0606(8)	—
1.7	α-Fe_2O_3	flat quartz	0.1278(17)	0.1118(19)
1.7	α-Fe_2O_3	flat Al	0.1208(18)	0.1044(20)
1.7	$CaCO_3$	flat Al	0.0705(9)	0.0360(17)
2.27	empty	flat Al	0.0560(21)	—
2.27	$CaCO_3$	flat Al	0.0596(6)	0.0203(18)
2.27	Fe_2O_3	flat Al	0.0827(21)	0.0541(32)
2.3	empty	flat quartz	0.0583(9)	—
2.3	α-Fe_2O_3	flat quartz	0.0878(6)	0.0656(9)
2.3	empty	cylinder	0.0479(3)	—
2.3	α-Fe_2O_3	cylinder	0.1514(4)	0.1439(6)

S2. $\alpha - Fe_2O_3$ (hematite)

Fig. S1 and Table S2 show additional LD data from α-Fe_2O_3 for the (116) and (214) reflections (for (024) see Fig. 7 in the main text). The contribution of SANS to the polarization decreases with increasing k, and is more pronounced for the cylindrical sample. After correction by the SESANS measured at $k = 1.7\,$Å^{-1} on the flat container (Fig. 5(a), main text), the agreement of the intrinsic polarization signal P_{LD} for both container types is satisfactory, where the data from the flat container are more reliable due to the smaller correction. Both SR and LD data show significantly broader widths (Table S2) than those calculated from the the monoclinic structure model established in ref. [2], although the widths follow the trend of the calculated values. The main contribution to the width is probably due to micro strains in the powder grains.

TABLE S2. Distribution of d_{hkl} in α-Fe_2O_3 for the flat quartz container, the cylindrical vanadium container, from SR diffraction, and calculation using a monoclinic structure model. All values in this table are in units of 1×10^{-4}.

(hkl)	ϵ_{hkl} flat	ϵ_{hkl} cyl.	width SR	width calc.
(024)	7.06(15)	7.10(40)	6.76(6)	2.22
(116)	6.32(15)	6.01(28)	6.19(5)	1.67
(214)	5.06(21)	4.91(30)	5.12(5)	0.95
(300)	5.53(21)	—	5.18(5)	1.12

We also tested the alignment of the flat Al container in the transmission and reflection geometries, which means setting the flat sample surface perpendicular (reflection) or parallel (transmission) to Q. As the effective path length in the flat sample of width t_0 at the Bragg angle θ is $L_r = t_0/\sin(\theta)$ in reflection and $L_t = t_0/\cos(\theta)$ in transmission, the ratio $L_r/L_t = \tan(\theta)$ is unfavorable for the transmission geometry for $\theta > 45°$. For the LD experiments we used $\theta = 55°$, such that $L_t/L_r \simeq 1.4$. This effective path length difference results in a larger Gaussian width of the LD raw data (without any SANS or other corrections). For the (116) reflection we get $\epsilon_{\mathrm{raw}} = 9.41(5) \times 10^{-4}$ in transmission and $8.68(5) \times 10^{-4}$ in reflection.

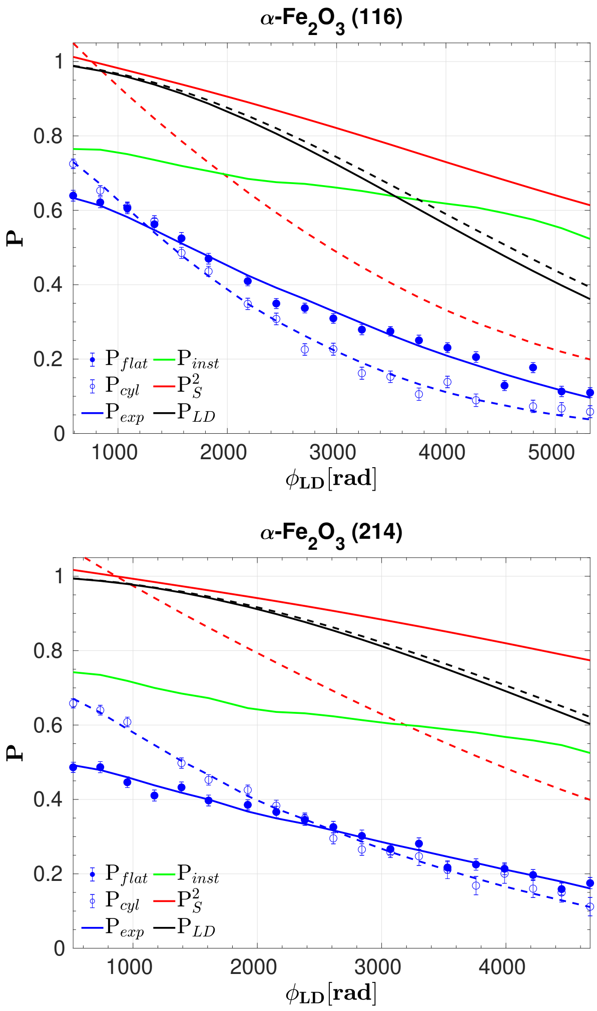

FIG. S1. Comparison of the Larmor diffraction signal from α-Fe_2O_3 for the flat (closed symbols, solid lines) and cylindrical (open symbols, dotted lines) sample containers for the (116) and (214) reflections in α-Fe_2O_3, measured at $k = 2.26\,\text{Å}^{-1}$ and $k = 2.57\,\text{Å}^{-1}$, respectively.

S3. $CaCO_3$ (calcite)

TABLE S3. Distribution of d_{hkl} in $CaCO_3$. Comparison of LD, SR, and calculation. All values in this table are in units of 1×10^{-4}.

(hkl)	LD ϵ_{hkl} flat Al	width SR	width calc.
(113)	5.78(7)	4.54(7)	1.64
(018)	6.20(15)	5.08(7)	1.71
(211)	5.06(12)	4.02(11)	1.0

Three Bragg peaks in $CaCO_3$ were studied by LD, where the scattering angle $2\theta = 110°$ was kept constant, and k was set to 1.68, 2.01, and $2.36\,\text{Å}^{-1}$ for the (113), (018), and (211) reflections, respectively. Fig. S2 shows the SESANS and LD polarization for these three peaks. As mentioned before, the SANS contributed significantly less to the LD polarization than in the case of α-Fe_2O_3. The comparison of the distribution widths with SR data from ID22 [1] is plotted in Fig. S3, the numbers are summarized in Table S3. The agreement of LD and SR data is less good than in the case of α-Fe_2O_3. There is no obvious reason for this discrepancy, both samples of $CaCO_3$ were taken from the same batch. Eventually environmental conditions such as humidity could have led to a change

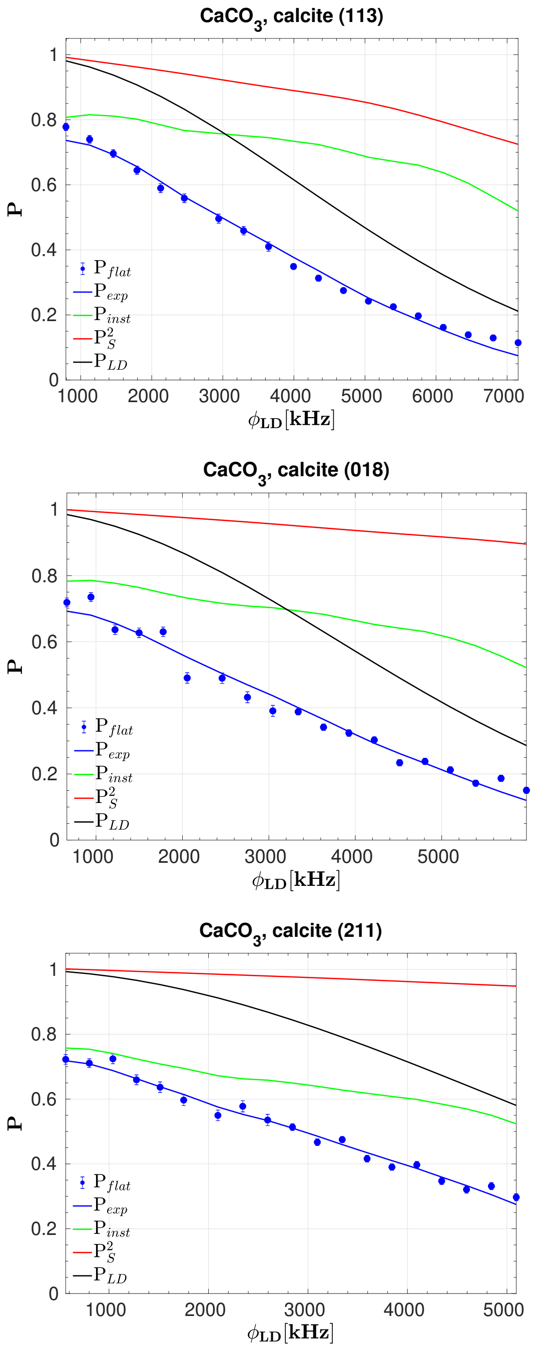

FIG. S2. Larmor diffraction data from $CaCO_3$ in a flat sample container (symbols) for the (113), (018), and (211) reflections, respectively.

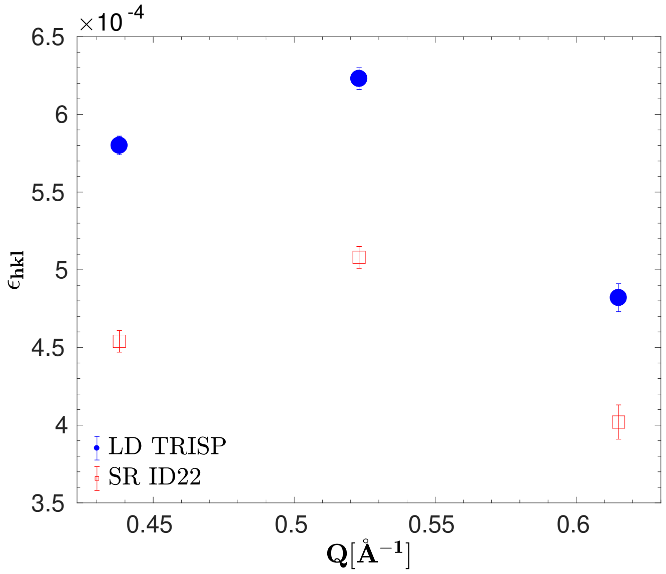

FIG. S3. $\Delta d/d$-spacing distribution ϵ_{hkl} (FWHM) *vs.* $Q = 1/d_{\mathrm{hkl}}$ of three Bragg peaks from $CaCO_3$, calcite, obtained from LD (flat container) and SR diffraction. The d-distribution widths ϵ_{hkl} are $5.80(6) \times 10^{-4}$, $6.23(9) \times 10^{-4}$, and $4.82(9) \times 10^{-4}$ for the (113), (018), and (211) reflections.

of the sample properties, as there was a time span of 9 years between the SR and the LD experiments. Both the SR and LD distribution widths are much broader than the width calculated from the monoclinic structure model established in ref. [1]. This means that the peak width is dominated by other mechanisms, such as micro strains. The variations of the peak widths, however, roughly follow the variations of the calculated values.

[1] R. Przeniosło, P. Fabrykiewicz and I. Sosnowska, Physica B **496**, 49-56 (2016).

[2] R. Przeniosło, I. Sosnowska, M. Stękiel, D. Wardecki, and A. Fitch, Physica B, **449**, 72-76 (2014).
